# Botryomycosis: A Case Series and Literature Review in Mexico

**DOI:** 10.7759/cureus.107815

**Published:** 2026-04-27

**Authors:** María Fernanda Limón Limón, Marco Antonio Rodríguez Castellanos, Guillermo Solís Ledesma, Guillermo Manuel Amezcua Rosas, Amara Hazel Solorio Rivera, Andrea Sierra Franco, Jorge Arturo Mayorga Rodríguez

**Affiliations:** 1 Dermatology, Instituto Dermatológico de Jalisco "Dr. José Barba Rubio", Zapopan, MEX; 2 Dermatology, Universidad de Guadalajara, Guadalajara, MEX; 3 Laboratory Medicine, Instituto Dermatológico de Jalisco "Dr. José Barba Rubio", Zapopan, MEX; 4 State Clinic for Oral Mucosal Diseases, Instituto Dermatológico de Jalisco "Dr. José Barba Rubio", Zapopan, MEX; 5 Reference Center in Mycology, Instituto Dermatológico de Jalisco "Dr. José Barba Rubio", Zapopan, MEX

**Keywords:** botryomycosis, cutaneous botryomycosis, grains, splendore-hoeppli phenomenon, staphylococcus aureus

## Abstract

Botryomycosis is a rare chronic bacterial infection caused by non-filamentous organisms, characterized by nodular or fistulous lesions with purulent exudate and the presence of grains that may mimic other granulomatous infections. The most commonly isolated causative agent is *Staphylococcus aureus*, although several other species have been reported. Because it may be mistaken for deep mycoses or neoplasms, diagnosis relies on histopathological examination, microbiological cultures with antimicrobial susceptibility testing, and, when available, molecular or proteomic identification methods.

Three cases of botryomycosis affecting different anatomical locations are presented, describing their diagnostic approach and the medical-surgical treatment employed. The importance of considering this entity in the differential diagnosis of chronic granulomatous dermatoses is emphasized. This case series also expands the available evidence on this disease in Mexico, where only 16 cases, including the present series, have been documented in the medical literature.

## Introduction

Botryomycosis is a chronic, suppurative, and granulomatous bacterial infection caused by non-filamentous Gram-positive or Gram-negative bacteria that are aerobic or microaerophilic and form grains (biofilm composed of masses of bacteria agglomerated by interstitial cement). It is an uncommon condition that primarily affects the skin and, on rare occasions, internal organs [1]. Its development is usually associated with multiple factors, generally following a lesion or surgical intervention [2].

The first case of botryomycosis was reported in 1870 by Bollinger in a horse that developed the disease as a complication following castration, with dissemination to the lungs [3]. In 1884, Rivolta proposed the term “botryomycosis,” derived from the Greek botrys (“grape cluster”), referring to the characteristic appearance of the granules, and mycosis, since it was initially considered a fungal disease [4]. The first human cases were reported between 1910 and 1913 by Archibald [5] and Opie [6]. Later, Magrou demonstrated its bacterial origin, identifying *Staphylococcus aureus* as the causative agent and establishing that the grains consisted of bacterial aggregates held together by a substance he termed “cement” [7].

Although the term botryomycosis is not the most appropriate, as it suggests a fungal rather than bacterial infection, it is the one that has been accepted in the literature. Other designations have also been employed to refer to this disease, such as staphyloactinomycesis, granular bacteriosis, bacterial pseudomycosis, and actinobacillosis [8].

*S. aureus* is the principal causative agent (40%), followed by *Pseudomonas aeruginosa* (20%). Other microorganisms identified with lesser frequency include *Micrococcus pyogenes*, coagulase-negative *Staphylococcus*, alpha-hemolytic *Streptococcus*, *Escherichia coli*, *Peptostreptococcus* sp., *Bacteroides fragilis*, *Pseudomonas cepacia*, and *Proteus vulgaris*. Also implicated are *Propionibacterium acnes*, *Neisseria* sp., *Serratia marcescens*, *Actinobacillus lignieresii*, *Pseudomonas vesicularis*, and *Moraxella nonliquefaciens* [8,9]. Recently, Yendo et al. reported a case of cutaneous botryomycosis caused by *Staphylococcus warneri*, diagnosed by matrix-assisted laser desorption/ionization time-of-flight (MALDI-TOF) in a healthy 58-year-old woman [9]. Likewise, the coexistence of two or more bacterial species in the same case has been documented, as reported by Rojas-Plasencia and Zapata-Granja, who isolated *S. aureus* and *Peptostreptococcus* sp. in one case, and *Actinobacillus* sp. and *S. aureus* in another [10].

The geographical distribution of this entity lacks clinical relevance, since the causative microorganisms, such as *S. aureus*, are part of the skin and mucous membrane microbiome and can be isolated in any region of the world [8]. Although some pathogens have been reported more frequently in cases of botryomycosis, it has not been determined whether this association is due to their prevalence, specific virulence factors, or other mechanisms involved in the pathogenesis of the disease [11]. Various hypotheses have been proposed to explain its development. In experimental studies, Magrou conclusively established the bacterial origin of botryomycosis by demonstrating that direct inoculation of *S. aureus* is sufficient to induce the disease. Furthermore, he observed a dose-dependent effect, where a low bacterial load is controlled by the host immunological response, whereas a high load favors abscess formation. These findings highlight the importance of the balance between the microorganism's virulence and the host immune response in its development [7]. Drake et al. proposed that low-virulence pathogens play a fundamental role in pathophysiology, a hypothesis supported by other studies [12,13]. The host's inability to generate an effective immunological response also represents a key factor. It is likely that the interaction among multiple elements, including the bacterial species, its virulence, the inoculum load, and the host immune response, determines whether the infection evolves into a case of botryomycosis or not [11].

Winslow clinically classified botryomycosis into two forms: cutaneous and visceral, with the former accounting for up to 75% of reported cases [1,14]. The infection can occur in both immunocompromised patients and immunocompetent individuals. Predisposing factors include diabetes mellitus, post-surgical complications, cutaneous trauma, liver diseases, prolonged steroid use, alcoholism, and cystic fibrosis [8]. It may present at any age, with a slight male predominance (3:2 ratio) [15]. The youngest documented case corresponds to an 8.5-month-old girl [16], while one of the oldest patients was 80 years old [17]. The cutaneous form can affect skin, subcutaneous tissue, muscle, and bone. The typical lesion consists of small painful nodules that drain purulent secretion; ulcerated, verrucous lesions or fistulas with seropurulent discharge may also be present, in which grains measuring 3 to 5 mm can be observed under microscopy. The extremities are the most common location, although cases have also been described on the trunk, face, and perianal region [18]. In the oral cavity, involvement of the tongue, tonsils, and palate has been reported [19]. Most cases are localized; however, the disease can disseminate and affect practically any part of the body [20]. Symptoms may fluctuate but tend to worsen with disease progression. Finally, patients seek medical attention due to painful exacerbations of chronic infection [21]. The differential diagnosis includes mycotic infections such as mycetoma, actinomycosis, and sporotrichosis, as well as verrucous tuberculosis, abscesses, among others [14].

Diagnosis is complex, particularly in early stages, due to clinical and histopathological similarity with chronic granulomatous diseases of fungal or actinomycetal origin, as well as with bone and soft tissue neoplasms, even on imaging studies [21]. In many cases, diagnosis is established through histopathological analysis of surgical samples or from retrospective reviews of documented cases. Sample collection is obtained from exudate of active ulcers or fistulas; fine-needle aspiration is useful, and in visceral cases, samples are obtained through biopsy. Diagnosis is established by direct examination with 20% potassium hydroxide (KOH) solution or Lugol's solution, where white-yellowish or "sulfur-colored" grains are observed, which are soft, 1 to 3 mm in size, non-filamentous, and similar to those of *Actinomyces israelii* and *Actinomadura madurae*. The presence of red grains caused by *S. aureus* has been reported, which must be distinguished from those produced by *Actinomadura pelletieri* [8,14].

Histopathology constitutes an effective method for diagnosing botryomycosis, as it allows evaluation of the morphology and staining characteristics of the grains, facilitating differentiation from entities such as actinomycosis and mycetoma. Typical histopathological findings show an epidermis with hyperkeratosis and acanthosis. Granulomas composed of neutrophils, lymphocytes, eosinophils, plasma cells, fibroblasts, histiocytes, and occasionally foreign body-type giant cells are identified in the dermis. The grains, approximately 1 mm in size, are amphophilic, lobulated, and non-filamentous, consisting of coccoid or bacillary elements arranged in clusters, and are located at the center of the inflammatory reaction. In some cases, the Splendore-Hoeppli phenomenon is observed, characterized by amorphous eosinophilic material surrounding them, forming a radiated halo that corresponds to a localized immunological response related to antigen-antibody complex formation. They are negative on Gomori-Grocott methenamine silver stain, which contributes to their differentiation from fungal etiologies [8,14,20].

Definitive diagnosis is established through culture, prior to exclusion of fungal and actinomycete infections. It is essential to obtain samples for culture on conventional media for aerobic and anaerobic bacteria [14,21].

Regarding the role of molecular biology, it can be useful for the precise identification of the microorganisms involved, especially in cases where conventional culture is negative or insufficient. Techniques such as PCR, multilocus sequencing to obtain regions of the 16S rRNA, rpoB, recN, rpoA, and tdhF genes, compared against genetic databases, will provide more precise taxonomic information on the etiological agents involved in the infection, thereby optimizing therapeutic and clinical management [22].

Treatment consists of antibiotic administration for several weeks, according to the causative agent and its susceptibility profile. Erythromycin (500 mg every six hours), minocycline (100 mg/day), trimethoprim/sulfamethoxazole (800/160 mg every 12 hours and in convenient doses), dicloxacillin, gentamicin, benzathine penicillin, cefazolin, and cefaclor have been successfully used. In most cases, surgical resection and drainage are required. The prognosis of the cutaneous form is usually favorable [8].

## Case presentation

Case 1

**Figure 1:**
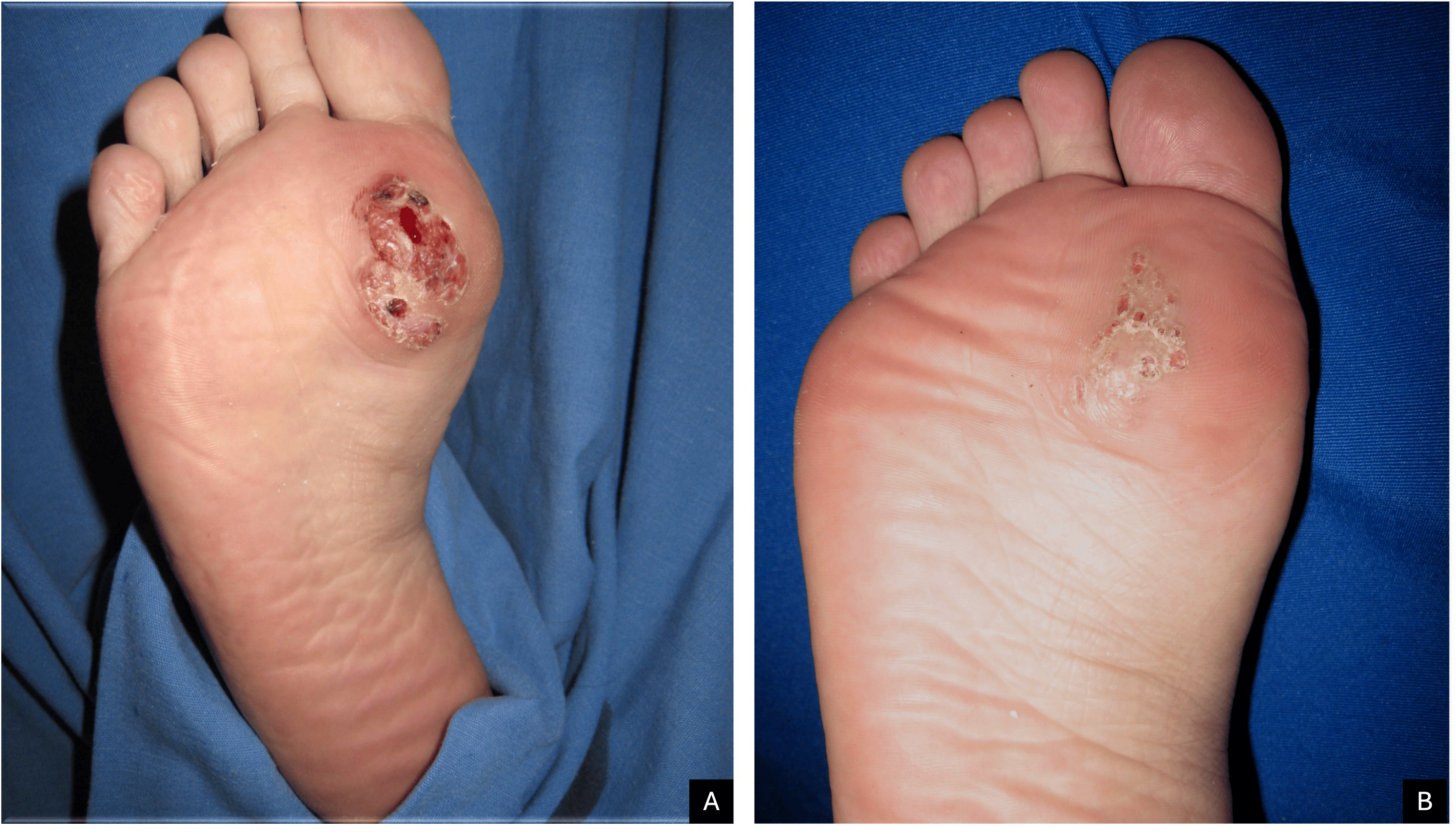
(A, B) Case 1

A 27-year-old woman with no relevant past medical history presented with a one-year history of a lesion located on the plantar surface of the right foot at the level of the first metatarsal. The lesion was localized and asymmetric, consisting of a vegetating verrucous mass measuring 5 × 3.5 × 0.5 cm. It showed areas of hyperkeratosis, mamillated projections, hemorrhagic foci, and hematic and honey-colored crusts. The borders were well defined, elevated, and keratotic. The lesion was normothermic, firm in consistency, tender to palpation, and infiltrated into deeper tissues (Figure 1A).

Case 2

A 62-year-old man with diabetes mellitus presented with stomatitis involving the soft and hard palate on the right side of the midline. Examination revealed a lobulated exophytic mass measuring 5 × 4 × 4 cm, with an erythematous surface and focal necrotic areas. The lesion had well-defined but infiltrated borders and was adherent to deeper structures, without bone involvement, suggesting chronic evolution.

**Figure 2:**
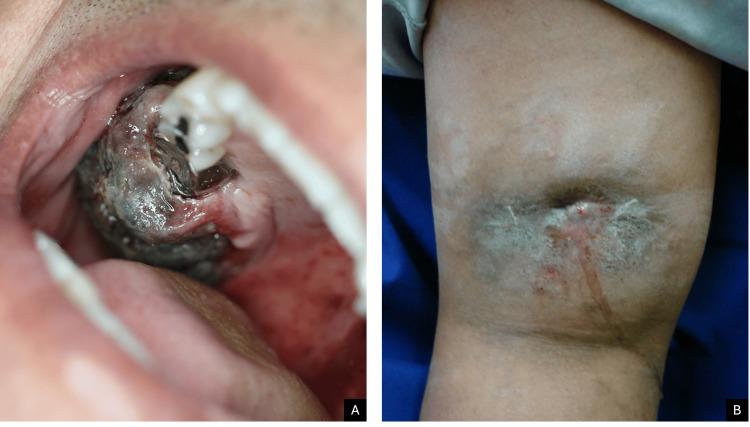
(A) Case 2. (B) Case 3

The patient reported a history of extraction of the right maxillary second molar. Three weeks after the procedure, exuberant granulation tissue developed at the surgical site, requiring local excision. However, the lesion recurred one month later, prompting surgical removal of the tumor (Figure 2A).

Case 3

A 17-year-old woman with no relevant past medical history presented with a two-year history of a lesion located on the posterior aspect of the left thigh. Physical examination revealed an ovoid plaque measuring 12 × 7 × 4 cm, erythematous and hyperpigmented, with a centrally ulcerated area draining purulent exudate. The surface was covered with serohemorrhagic crusts, and post-inflammatory hyperpigmentation was observed in the surrounding skin. The lesion had poorly defined borders and was infiltrated on palpation (Figure 2B).

An incisional biopsy was performed in all three cases. Histopathological examination revealed an acanthotic epidermis with focal ulceration. Within the dermis, dilated blood vessels and a dense inflammatory infiltrate composed of lymphocytes, histiocytes, and neutrophils were observed.

**Figure 3:**
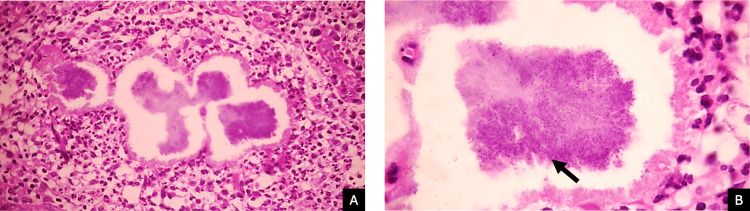
Histopathological examination showing suppurative granulomatous inflammation with bacterial granules. (A) Low-power view demonstrating dermal inflammatory infiltrate surrounding the lesion (H&E, 10×). (B) Higher magnification highlighting clusters of coccoid bacteria (black arrow) surrounded by eosinophilic material consistent with the Splendore-Hoeppli phenomenon (H&E, 40×)

At higher magnification, neutrophilic microabscesses containing amphophilic granules measuring 200-500 μm in diameter were identified. These granules consisted of clusters of coccoid bacteria surrounded by an eosinophilic material forming the Splendore-Hoeppli phenomenon, findings consistent with botryomycosis (Figures 3A, 3B).

**Figure 4:**
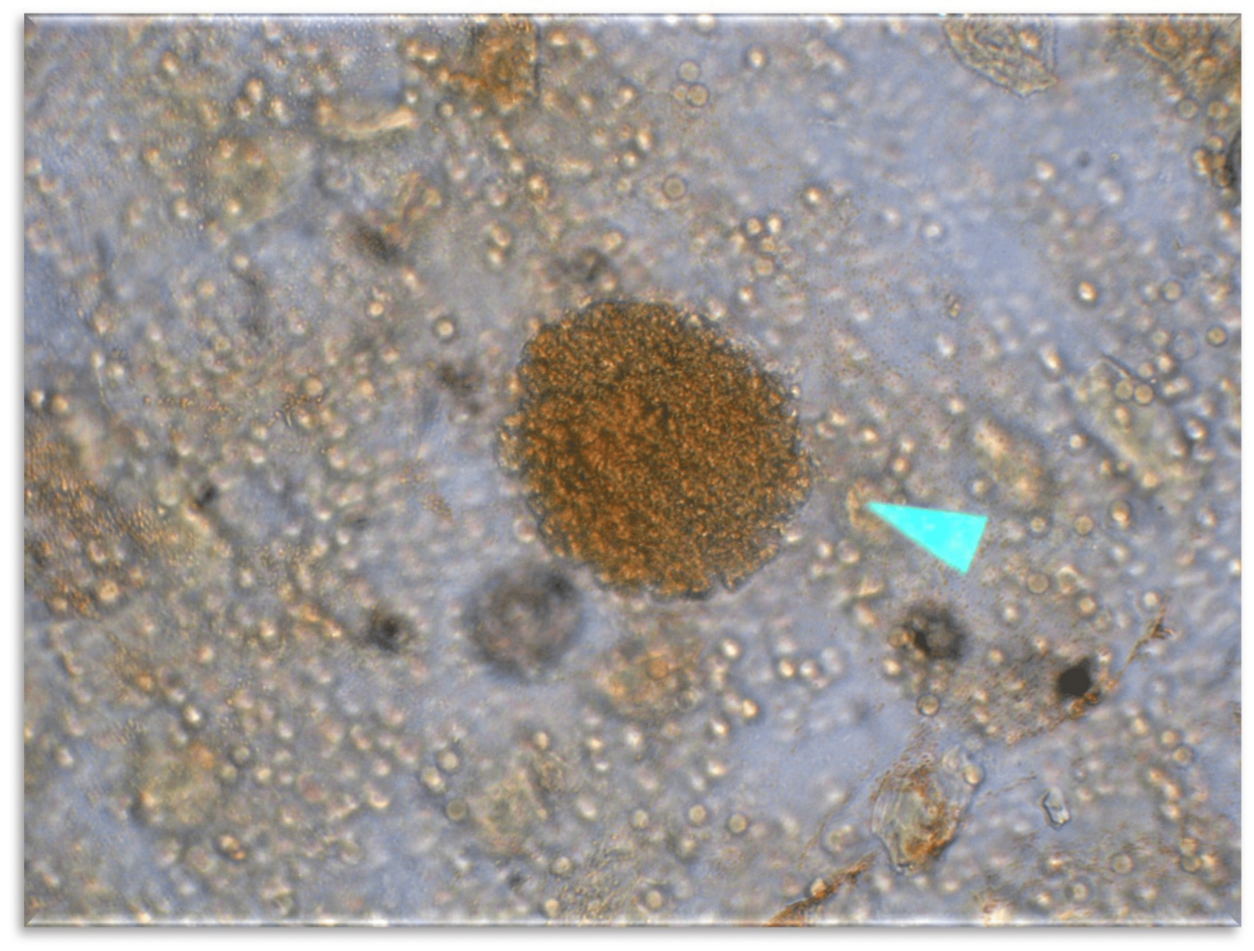
Direct examination with Lugol’s solution showing bacterial granules

Direct examination with Lugol’s solution demonstrated granules composed of bacterial aggregates (Figure 4). Gram staining revealed Gram-positive cocci compatible with *Staphylococcus* species.

Culture on blood agar yielded small yellow colonies after three days of incubation. Identification using the bioMérieux VITEK® 2 automated system (Marcy-l'Étoile, France) confirmed *S. aureus*. Antimicrobial susceptibility testing demonstrated sensitivity to dicloxacillin, which was administered orally at 500 mg every six hours in all three patients. Complete clinical resolution was achieved after three, four, and 12 weeks, respectively (Figure 1B), with no recurrence after two years of follow-up.

## Discussion

A literature search was conducted to identify previously reported cases of cutaneous botryomycosis in Mexico using the keyword “cutaneous botryomycosis” across the databases PubMed, Medigraphic, and Revista Mexicana de Dermatología, covering the period from January 1, 1995, to December 1, 2025. Thirteen cases were identified in the literature. When combined with the three cases described in the present study, a total of 16 cases have been reported in Mexico, including two cutaneous cases and one involving the oral mucosa in the current series.

Among the 16 reported cases, the age ranged from seven to 76 years, with a mean age of 36.06 years. The most frequently affected anatomical site was the lower extremities, accounting for eight of 16 cases (50%). *S. aureus* was isolated in 10 cases (62.5%), which is consistent with previous reports identifying this organism as the most common etiologic agent of botryomycosis.

All patients showed a favorable clinical response to antibiotic therapy. The most frequently used regimen was trimethoprim-sulfamethoxazole, followed by dicloxacillin, resulting in complete resolution of the lesions without evidence of recurrence during follow-up. In 1995, Moreno Collado reported seven cases, representing the largest case series published in Mexico to date (Table 1).

**Table 1:** Reported cases of cutaneous botryomycosis in Mexico identified through a literature review (1995-2025) TMP/SMX: trimethoprim/sulfamethoxazole

Author/year	Age/sex	Site	Causative agent	Treatment	Outcome
Moreno Collado, 1995 [23]; 7 cases	7 years; male	Left ankle	Microbiological studies were not performed	Surgical excision	No recurrence at 6 months
	29 years; male	Left paraumbilical region	Microbiological studies were not performed	Surgical excision	Complete resolution
	70 years; male	Abdomen	Pseudomonas aeruginosa	Gentamicin/amikacin + TMP/SMX	Clinical resolution at 2 months
	56 years; female	Abdomen	Enterobacter agglomerans	TMP/SMX	Clinical resolution at 2 weeks
	41 years; female	Left arm	Staphylococcus aureus	Ampicillin	Clinical resolution at 3 weeks
	19 years; male	Left palm	Staphylococcus aureus	Drainage + unspecified antibiotic therapy	Clinical resolution at 1 month
	76 years; female	Left dorsal hand	Staphylococcus aureus	Fusidic acid + ampicillin	Clinical resolution at 1 week
Cubilla et al., 2010 [24]; 2 cases	13 years; male	Left foot	Staphylococcus aureus	TMP/SMX	No recurrence at 3 months
	38 years; male	Periumbilical region	Staphylococcus aureus	Ciprofloxacin	Clinical improvement
Villanueva Otamendi et al., 2012[25]; 1 case	23 years; male	Right leg	Klebsiella pneumoniae	Ciprofloxacin + rifampin + TMP/SMX	Complete resolution
Flores et al., 2015 [26]; 1 case	44 years; male	Gluteal and perineal regions	Staphylococcus aureus	TMP/SMX	
Ocampo-Garza et al., 2017 [27]; 1 case	20 years; male	Left foot	Pseudomonas fluorescens	Ceftazidime	No recurrence at 8 months
López-Jiménez et al., 2024 [28]; 1 case	35 years; female	Head, trunk, lower extremities	Staphylococcus aureus	Dicloxacillin	Complete resolution
Limón Limón et al., 2025; 3 cases	27 years; female	Right foot	Staphylococcus aureus	Dicloxacillin	Clinical resolution at 3 weeks
	62 years; male	Oral cavity	Staphylococcus aureus	Dicloxacillin	Clinical resolution at 4 weeks
	17 years; female	Left thigh	Staphylococcus aureus	Dicloxacillin	Clinical resolution at 3 months

## Conclusions

Botryomycosis is a rare chronic suppurative-granulomatous bacterial infection that represents a diagnostic challenge due to its clinical and histopathological similarity to other conditions. Diagnosis requires a high index of suspicion and confirmation through histopathology-highlighting the Splendore-Hoeppli phenomenon-and microbiological studies. In Mexico, 16 cases have been reported over the past 31 years, predominantly affecting the skin, especially the lower extremities, with *S. aureus* as the main etiologic agent, consistent with international data.

Targeted antibiotic therapy, often combined with surgical management, leads to favorable outcomes. In our series, all patients responded successfully to oral dicloxacillin. This review highlights the likely underdiagnosis of botryomycosis in Mexico and Latin America and underscores the importance of including it in the differential diagnosis of chronic verrucous, nodular, or ulcerated lesions with purulent discharge and granules to avoid delayed diagnosis and inappropriate treatment.
